# A Randomized Double-Blind Placebo-Controlled Trial to Evaluate Prophylactic Effect of Traditional Chinese Medicine Supplementing Qi and Hemostasis Formula on Gastrointestinal Bleeding after Percutaneous Coronary Intervention in Patients at High Risks

**DOI:** 10.1155/2018/3852196

**Published:** 2018-10-21

**Authors:** Chenhao Zhang, Chaolian Huang, Xiaolin Kong, Guannan Liu, Ning Li, Jie Liu, Zongyao Zhang, Dawei Yang, Chunling Liang, Jie Wang

**Affiliations:** ^1^Wangjing Hospital, China Academy of Chinese Medical Sciences, Beijing 100102, China; ^2^Guang'anmen Hospital, China Academy of Chinese Medical Sciences, Beijing 100053, China

## Abstract

**Objective:**

To evaluate the clinical efficacy of traditional Chinese medicine (TCM) supplementing Qi and hemostasis formula on gastrointestinal (GI) bleeding after percutaneous coronary intervention (PCI) and thus find out the optimal therapeutic regimen to reduce incidence of GI bleeding without increase of major adverse cardiovascular events (MACEs).

**Methods:**

In the randomized, double-blinded, controlled trial, 117 participants who underwent PCI were enrolled and evenly distributed into treatment arm (59) and control arm (58). Numerous end points were assessed including the primary endpoint of GI bleeding and MACEs and secondary endpoint of thromboelastogram (TEG) (mainly MAadp, inhibition of ADP, and inhibition of AA) and TCM syndrome score during the follow-up phase of 90 days.

**Results:**

Incidence of bleeding including GI bleeding and MACE did not differ significantly between two arms (28.82% in treatment arm versus 24.44% in control). However, on both days 30 and 90, TCM treatment remarkably reduced the TCM syndrome total score with notable alteration (P<0.05) except for some parameters such as pulse manifestation. When it came to TEG, however, MA_ADP_ increased significantly on day 30 in control arm, accompanied by a notable descending in inhibition rate of ADP pathway (both P<0.01).

**Conclusion:**

(1) Supplementing Qi and hemostasis formula is equal to Pantoprazole Sodium Enteric-Coated Capsule in hemostasis and gastric mucosal protection; (2) supplementing Qi and hemostasis formula is superior to Pantoprazole Sodium Enteric-Coated Capsule in improving TCM syndrome manifestation possibly through the multitarget mechanism; (3) interference on clopidogrel of supplementing Qi and hemostasis formula might be much less than Pantoprazole Sodium Enteric-Coated Capsule due to the potential CYP450-independent mechanism. This trial is registered with ChiCTR1800014485.

## 1. Introduction

With the development of percutaneous coronary intervention (PCI) and the regular use of dual-antiplatelet drugs, ischemic events after PCI has been remarkably reduced, whereas gastrointestinal (GI) bleeding in patients at high bleeding risks is gradually becoming a major concern. The application of proton pump inhibitors (PPI) after PCI in order to prevent GI bleeding is controversial due to the competitive inhibition between PPI and clopidogrel that occurs at CYP2C19 iso-enzyme and a potential compromise of platelet inhibition by clopidogrel which might be associated with PPI use. We, therefore, based on our previous work, conducted the current prospective randomized, double-blinded, controlled clinical trial to investigate the prophylactic effect of traditional Chinese medicine (TCM) supplementing Qi and hemostasis formula on GI bleeding as well as major adverse cardiovascular event (MACE) incidence after PCI, aiming to optimize the benefit of TCM to prevent GI bleeding and simultaneously attenuate MACE recurrence.

## 2. Materials and Methods

### 2.1. Patients

124 patients underwent PCI in Cardiovascular Intervention Center of Wangjing Hospital during May 2016 to May 2017 and presenting Qi deficiency and blood stasis syndrome were included. After exclusion of patients that died or quitted from the protocol, 117 participants were finally recruited. Participants enrolled were randomized in a 1:1 fashion to either treatment arm (n=59) or control arm (n=58).

### 2.2. Diagnostic Criteria

#### 2.2.1. Diagnostic Criteria for CHD

ST-elevated myocardial infarction (STEMI) was diagnosed according to the 2010 Guideline for the diagnosis and treatment of acute ST-elevated myocardial infarction published by Cardiovascular Committee, China Medical Association [1]. Diagnosis of unstable angina pectoris (UAP) and non-ST-elevated myocardial infarction (NSTEMI) referred to 2012 Guideline for the diagnosis and treatment of non-ST-elevated myocardial infarction published by Cardiovascular Committee, China Medical Association [2]. Stable angina pectoris (SAP) was diagnosed according to the 2007 Guideline for the diagnosis and treatment of chronic stable angina pectoris published by Cardiovascular Committee, China Medical Association [3].

#### 2.2.2. Diagnostic Criteria for GI Bleeding after PCI

Newly occurring significant hematemesis or melena, hematocrit (HCT) reduced by ≥15%, and/or hemoglobin decreased by ≥30g/L after PCI compared with before operation [4].

#### 2.2.3. Definition of MACE

1. Cardiac death: cardiac death featured abrupt unconsciousness within 1 hour of acute symptom attack.
2. Nonfatal recurrent myocardial infarction: recurrent chest pain or newly occurring electrocardiograph (ECG) change, accompanied by twofold increase in myocardial enzyme; for patients whose elevated myocardial enzyme had not recovered, recurrent increase in myocardial enzyme is the indication.
3. Recurrent UAP: relapsing or deterioration of symptoms of cardiac ischemia, exacerbated ST segment depression, or the need to increase dose of nitrates to alleviate symptoms.
4. Subacute in-stent thrombogenesis: angiography documented in-stent restenosis within 24 hours to 30 days after PCI and TIMI blood flow 0-1 class.
5. Revascularization of targeted vessel: revascularization of coronary that is related to prior infarction including CABG and PCI.

### 2.3. Inclusion Criteria

1. Meet the diagnostic criteria of CHD.
2. Underwent PCI operation successfully.
3. CRUSADE score ≥41.
4. Be able to sign informed consent.

### 2.4. Exclusion Criteria

1. Use of Warfarin or other agents that might affect platelet aggregation within two weeks.
2. Platelet number <100 × 10^9^/L.
3. Anemia, hemoglobin <100g/L.
4. Active peptic ulcer.
5. Use of PPI within one month.
6. Bleeding tendency or hematological disorders.
7. Pregnant and lactating female.
8. Allergic to component of the test drugs and allergic constitution.
9. Participated to other clinical trial within one month.

### 2.5. Randomization

Randomization was generated using random permuted blocks. SAS 9.2 software PROC PLAN was applied to generate random number table for 124 patients included with the block length setting as 4. Patients were then randomized in a 1:1 fashion into two arms (treatment arm and control arm).

### 2.6. Medication

Composition of supplementing Qi and hemostasis formula: Astragalus 30 g, radix pseudostellariae 15 g, charred Radix et Rhizoma Rhei 15 g, rhizoma bletillae 15 g, cuttlebone 15 g, and pseudoginseng 3 g. Herbs were provided by Wangjing Hospital Pharmacy Department. Pantoprazole Sodium Enteric-Coated Capsule (40mg/capsule) was obtained from Hangzhou East China Pharmaceutical Company.

### 2.7. Protocol

In treatment arm, patients were given supplementing Qi and hemostasis formula and placebo matching with Pantoprazole Sodium Enteric-Coated Capsule. TCM formula was given in the form of granules and taken orally twice daily; Pantoprazole Sodium Enteric-Coated Capsule placebo was taken orally each morning and night on an empty stomach, whereas controlled arm was administered supplementing Qi and hemostasis formula placebo and Pantoprazole Sodium Enteric-Coated Capsule in the same manner with treatment group. All agents were administered for four weeks. During the follow-up phase, any agent preventing GI bleeding was prohibited.

### 2.8. Concomitant Treatments

All patients enrolled received concomitant standard therapy of PCI. Patients planning urgent PCI received loading dose clopidogrel (300mg)(Plavix, Sanofi-Aventis) and chew an aspirin (300mg) (Aspirin, Bayer) before operation; patients undergoing selective PCI started to take aspirin (100mg) and clopidogrel (75mg) orally three days prior to operation. All PCI operations enrolled in the investigation used drug-eluting stents. Dalteparin sodium (Pfizer, USA) (5000u, subcutaneous injection for 5-7 days) or GPIIb/IIIa receptor antagonist (Tirofiban, orally for 36-48 hours, Yuanda Pharmaceutical, Wuhan, China) was left to the discretion of investigator with consideration of patients' weight and state of illness. After PCI, clopidogrel (75mg) was administered continuously for 12 months, and aspirin (100mg) was recommended to taken as long-term medication. In addition, investigators were encouraged to follow the established guideline for CHD secondary prevention as to prescription of ACEI, *β*-receptor blockers, and statins.

### 2.9. Dual-Antiplatelet Therapy Counseling Programme

Adherence to dual-antiplatelet therapy (DAPT) is of vital importance. We therefore introduced the systematic DAPT counseling programme into our research with slightly moderation of the procedure reported by Simone Biscaglia et al. [5]. The major processes include the following:

(1) Short meetings (generally 15-minutes) initiated by the investigator focused on risks and advantages of DAPT, as well as the importance of adherence to DAPT 24 hours prior discharge with both participant and his/her kin.

(2) Brochure distribution stressing DAPT management.

(3) DAPT management brochure distribution to the patient's general practitioners and coordination with them.

(4) Keep available via a fixed phone number in case of any moderation of patient's DAPT therapy.

(5) Follow up through telephone twice per month to assess DAPT compliance.

### 2.10. Blindness

Either drugs or placebos were packaged in the same manner with the same label, the same color, and smell. According to the protocol design, blindness evidence was duplicated and sealed individually, reserved in investigation principal workplace and data analyzing workplace. Unblinding was processed in two-step manner: one after data entry and the other after data analysis. Investigators had access to information that could identify individual participants.

## 3. Clinical Data Collecting

### 3.1. General Clinical Data

Participants' age, gender, height, weight, ethnicity, and occupation were recorded. Other factors that might influence the efficacy were also recorded including medical history, admitting diagnosis, CRUSADE score, number of stent implantation, number of diseased vessels, part of diseased vessels, IABP implantation, and temporary pacemaker implantation.

### 3.2. Clinical Efficacy Endpoints

Clinical efficacy endpoints were evaluated within the follow-up phase of 90 days after operation. Primary endpoints included GI bleeding (BARC score was applied to evaluate the severity of bleeding) and MACE. Secondary endpoints included thromboelastogram (TEG) assessment of MA_ADP_, inhibition of ADP pathway and inhibition of AA pathway at day 7 and day 30; and TCM syndrome score after operation was evaluated at operation day, day 7, day 30, and day 90.

### 3.3. Safety End Points

1. blood routine test, urine routine test, and stool routine test.
2. Liver function (AST, ALT), kidney function (Cr, BUN), and coagulation function (PT, APTT, TT, and FIB).

### 3.4. Quality Control

Investigator brochure was published before the investigation beginning for training of all investigators. To guarantee the accuracy of data, data entry was processed by at least two data administrators after consistency check and revision of any inconsistent data until the information was accurate. Two experienced physicians were designated as guide for quality control and responsible for the supervision of data collecting process, data check, and data quality control.

## 4. Statistics

SAS 9.2 software was applied. All statistical tests were performed using two-sided hypothesis tests at the level of *α*=0.05 (*P*≤0.05 indicates statistical significance). Specific principles are as follows:

1. Quantitative data was described with mean and standard deviation. T-test (normal distribution; equal variance) or Wilcoxon rank-sum test (nonnormal distribution; equal variance not assumed) was used between groups; T-test or paired rank-sum test was used within group.
2. Qualitative data was described with frequency and percentage and analyzed using chi-square test or Fisher test. Rank data was analyzed using Wilcoxon rank-sum test.

### 4.1. Sample Size Calculating

Sample size in the current study was calculated based on the improvement of TCM syndrome score at day 30 after PCI operation. The formula was as follows [6]:

(1) $$\begin{array}{c} n_{1}=n_{2}=2{\left[\;\frac{\left(t_{1-\alpha /2}+t_{1-\beta }\right)S}{\delta }\;\right]}^{2} \end{array}$$

*δ* was the difference between means of two populations; S was the standard deviation. With reference to published literature and prior retrospective clinical trial data, *δ* was setting as 8 and S was 14.5. *α*=0.05, 1-*β* was 80%, and the sample size was estimated 108 using SAS 9.2 software. Considering the highest trial quitting rate 15%, the final size was estimated as 124.

## 5. Results

124 patients signed the informed consent at the enrollment phase and were randomized into treatment arm (62 patients) and control arm (62 patients). During the follow-up, 1 patient in the treatment arm died and 2 patients from the treatment arm quitted, whereas in the control arm, 4 patients quitted.

### 5.1. Demographic Characteristics

117 participants were finally enrolled in the study. The average age of treatment arm was 71.83 years old, 22 males (37.29%), and 37 females (62.71%), whereas the control arm had an average age of 71.14 years old, 20 males (34.48%) and 38 females (65.52%). Demographics were comparable between the two arms and showed no significant difference (P>0.05) as shown in Table 1.

### 5.2. Medical History

Prior diseases such as hypertension, type-2 diabetes mellitus (type-2 DM), lipid disorder, chronic heart dysfunction, chronic kidney dysfunction, chronic bronchitis, anemia, prior cerebrovascular diseases, peptic ulcer and prior smoking, and alcohol-intake history are distributed evenly between the two arms (*P*>0.05) as listed in Table 2.

### 5.3. Admitting Diagnosis and Operation

With regard to admitting diagnosis, 13 patients presented STEMI (23.21%), 14 were NSTEMI (23.21%), 20 were UAP (35.71%), and 9 were SAP (16.07%) in treatment arm, whereas control arm had 12 STEMI (21.43%), 12 NSTEMI (21.43%), 20 UAP (35.71%), and 12 SAP (21.43%). Two arms were comparable and showed no significant difference (*P*>0.05).

In terms of CRUSADE score, treatment arm got the average score of 56.51, whereas control arm was 54.66. There was no significant difference between the two (*P*>0.05).

When it comes to the number of stent implantation, treatment arm was 2.37 in average, whereas control arm was 2.19 and showed no significant difference (*P*>0.05).

Data about admitting condition, PCI puncture access, number of diseased vessel, part of diseased vessel, IABP implantation, and temporary cardiac pacemaker implantation was evenly distributed between the two arms (*P*>0.05) as shown in Table 3. 

## 6. Primary Endpoint

### 6.1. Incidence of MACE

**Figure 1:**
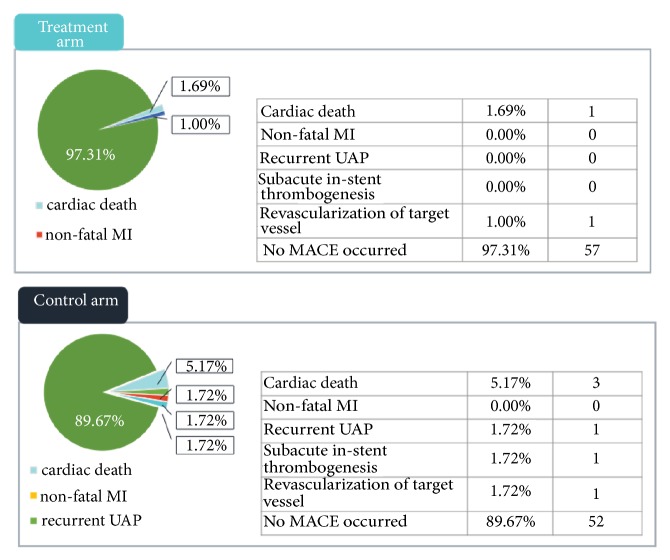
Incidence of MACE.

Two cases of cardiac event occurred in treatment arm after PCI (3.39%), of which one patient died due to cardiac causes, and the other received revascularization; 6 cases of cardiac events occurred in control arm (10.34%), including 3 cardiac death, 1 recurrent UAP, 1 subacute stent thrombogenesis, 1 revascularization of target vessel. As compared to control arm, incidence of MACE in treatment arm had a reduction by 6.95%, showing an obvious descending trend, although no significant difference showed between the two arms (*P*>0.05) as shown in Figure 1.

We analyzed the demographics between patients with ischemic event occurrence after PCI and patients without ischemic event occurrence. The result showed no significant difference in terms of age, gender, weight, height, SBP, HR, smoking, alcohol intake, CRUSADE score, admitting condition, admitting diagnosis, medical history, number of diseased vessel, part of diseased vessel, puncture site, IABP implantation, and TEG (Table 4).

### 6.2. Bleeding Events

Bleeding events occurred in 17 patients in treatment arm (28.81%). With reference to BARC bleeding classification, 11 were class 1 (64.71%), 5 were class 2 (29.41%), and 1 was class 3 (5.88%). In terms of bleeding site, 4 patients suffered GI bleeding (23.53%), 8 puncture site bleeding (7.06%), 1 urinary tract bleeding (5.88%), 1 intracranial bleeding (5.88%), 1 hemoptysis (5.88%), and 2 gingiva bleeding (11.76%).

**Figure 2:**
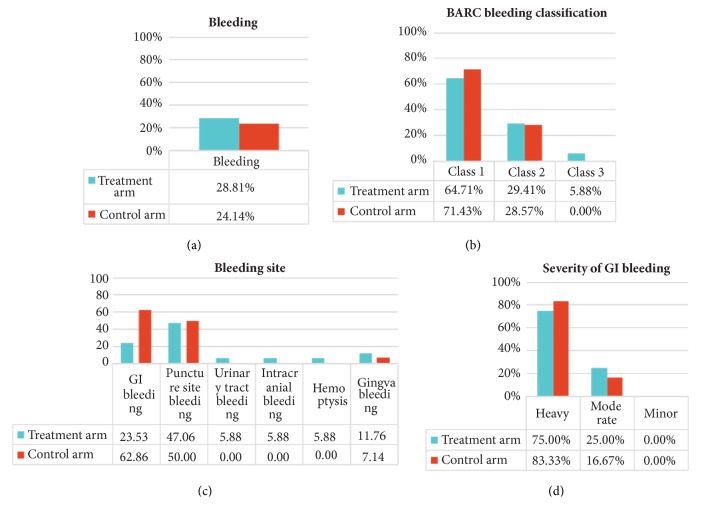
Bleeding occurrence in two arms.

With regard to control arm, 14 patients suffered postoperation bleeding (24.14%). According to BARC bleeding classification, 10 were classified as class 1 (71.43%), 2 were class 4 (28.57%), and none of the patient presented class 3. With regard to bleeding site, 6 were GI bleeding (42.86%), 7 were puncture site bleeding (50.00%), gingiva bleeding occurred in 1 participant (7.14%), no urinary tract bleeding, intracranial bleeding, or hemoptysis occurred. No significant difference was found between the two arms (*P*>0.05) as shown in Figure 2.

## 7. Secondary Endpoint

### 7.1. TCM Syndrome Score

On the operation day, symptoms including chest pain, chest tightness, palpitation, shortness of breath, debilitation, and signs including dark and purple lip, tongue, and pulse manifestation were evenly distributed between two arms and were comparable (P>0.05).

On day 7, patients from both arms reported significant alleviation in chest pain and chest tightness (*P*<0.05) and slight improvement in other symptoms (*P*>0.05). No significant difference was found between the two arms (*P*>0.05).

On day 30, symptoms and TCM syndrome score were improved significantly (P<0.05) except for the pulse score of control arm (P>0.05). It is noteworthy that the degree of improvement in treatment arm was much more superior to that of control arm, with statistically significant difference (P<0.05).

On day 90, symptoms and TCM syndrome score were improved significantly (P<0.05) except for the tongue and pulse manifestation of control arm (*P*>0.05). Degree of improvement in treatment arm was much more superior to that of control arm, with significant difference in terms of palpitation, shortness of breath, tongue manifestation, and total score (*P*<0.05) as shown in Table 5.

### 7.2. TEG

On day 7, data of MA_ADP_, inhibition rate of ADP pathway and inhibition rate of AA pathway was distributed evenly (*P*>0.05) and was comparable between two arms.

**Figure 3:**
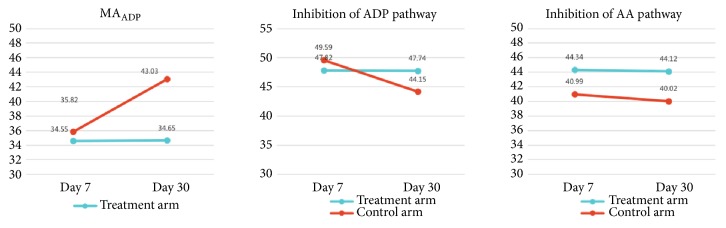
TEG in both arms. Note: ^△^on day 0, P value indicated comparison between the 2 arms; on days 7, 30, and 90, P value indicated the degree of improvement after treatment. *∗* indicated statistically significant difference between pre- and posttreatment.

Interestingly, as compared with day 7, MA_ADP_ on day 30 was significantly increased in control arm (*P*<0.05), whereas the inhibition rate of ADP pathway reduced remarkably (*P*<0.05 = as is shown in Figure 3.

## 8. Discussion

The application of dual-antiplatelet therapy subjected CHD patients especially those at high risk to the hazard of GI bleeding. Competitive inhibition at enzyme CYP2C19 between PPIs and clopidogrel compromises the effect of clopidogrel, inducing adverse cardiac events such as stent thrombogenesis [7]. Simultaneously, reduction of blood supplication subsequent to GI bleeding further exacerbated cardiac ischemia, and the vicious circle formed thereafter [8]. Imbalance between ischemia and bleeding after GI bleeding compromised the efficacy of PCI [9]. As a result, the current investigation focused right on the confliction of GI prevention.

There are three key findings in the present study: (1) supplementing Qi and hemostasis formula is not inferior to Pantoprazole Sodium Enteric-Coated Capsule in GI bleeding prevention; (2) the interference on the antiplatelet effect of clopidogrel from supplementing Qi and hemostasis formula might be much less than Pantoprazole Sodium Enteric-Coated Capsule, which was showed from the descending trend of MACE and comparison of TEG; (3) supplementing Qi and hemostasis formula is superior to Pantoprazole Sodium Enteric-Coated Capsule in improving TCM syndrome manifestation such as palpitation and shortness of breath.

### 8.1. Theory of Supplementing Qi and Hemostasis Formula

GI bleeding was categorized into bleeding syndrome of TCM. In TCM theory, Qi governs the blood: deficiency of Qi compromises the governing and leading effect of blood, which presents as blood extravasation from vessel or “bleeding”. GI bleeding after PCI has its specific pathology: firstly, the mechanical opening of the stenosis or occlusive vessel resembled “breaking blood” of TCM, which consumed Qi [10]; secondly, patients after PCI always suffered from anxiety, which leaded to spleen injury [11]; physical exercise loss after operation, particularly for those with MI who need to be bedfast, GI motion was decreased significantly, which leaded to malfunction of spleen[12]. The governing of Qi on blood was mainly associated with leading of spleen on blood. Vigorous spleen and sufficient Qi and blood enhances the governing of Qi and prevents extravasation of blood; on the contrary, malfunctioned spleen and deficient Qi and blood jeopardizes the governing of spleen, and thus induces bleeding. Therefore, GI bleeding after PCI was associated with Qi and spleen deficiency after operation and dysfunction of spleen in governing blood.

The TCM pathogenesis after PCI can be generalized as asthenia in origin and excess in superficiality, with Qi deficiency and blood stasis [13]. Researches focused syndromes prior to or after PCI indicated that “Qi deficiency and blood stasis” was the major TCM syndrome after PCI [14–17]. A retrospective investigation enrolling 801 patients underwent PCI indicated that Qi deficiency and blood stasis syndrome was correlated with GI bleeding; Qi deficiency and blood stasis syndrome together with gender, age, systolic pressure, Ccr were risk factors of GI bleeding [18]. Other investigators asserted that GI bleeding after PCI and enhanced dual-antiplatelet therapy should be attributed to Qi deficiency of spleen, as spleen Qi deficiency compromised the resistance towards outer harm and thus became susceptible to gastric ulcer [19].

Based on the above theory, we enrolled patients who underwent PCI and at high risk of GI bleeding, and supplementing Qi and hemostasis formula was administrated. The supplementing Qi and hemostasis formula in the current study contains Astragalus, radix pseudostellariae, charred Radix et Rhizoma Rhei, rhizoma bletillae, cuttlebone, and pseudoginseng. In clinical settings, the combination of the above herbs was frequently used for GI bleeding including stress ulceration and bleeding [20–24].

### 8.2. Supplementing Qi and Hemostasis Formula Prevents GI Bleeding

The current study showed that bleeding incidence, BARC score, and bleeding site did not differ significantly between two arms(P>0.05). With respect to GI bleeding, the incidence between two arms was similar, which indicated that the formula was not inferior to Pantoprazole Sodium Enteric-Coated Capsule in hemostasis and gastric mucosal protection.

From perspective of TCM herbs compatibility in a formula, Astragalus with the ability of supplementing Qi, generating and governing blood, served as the monarch drug. Charred Radix et Rhizoma Rhei and Pseudoginseng activated blood and removed blood stasis, thus serving as ministerial drug, assisted by radix pseudostellariae (supplementing Qi and improving spleen, nourishing Yin and generating jin), rhizoma bletillae (astringent and hemostasis) and cuttlebone (neutralizing gastric acid, hemostasis and generating tissues). All herbs interplays and synergizes with each other to achieve efficacies of supplementing Qi, governing blood, hemostasis and removing stasis, nourishing blood and generating tissues. Our present study showed that TCM herbs were superior to the control arm in improvement of TCM symptoms, implying the involvement of multitarget mechanism of action of TCM formula.

Astragalus is frequently used in TCM with the ability to supplementing Qi and generating blood. The major active subtract Astragaloside protected gastric mucosal through H^+^ K^+^-ATP enzyme and pepsin inhibition, gastric acid excretion reduction, and alleviation of stimulation of gastric mucosa [25]. Astragalus has antiulcer effect, which is associated with the component P8, P9, P12 [26]. Additionally, according to TCM theory, Astragalus exerts its action in blood, stops bleeding without blood stasis, and that is the reason for its common use in TCM. Besides, Astragalus relieves constipation by purgation, removes blood stasis and stops bleeding, which resembles GI decompression in modern medicine. Astragalus showed clearance effect of oxygen free radical which plays a vital role in intestinal mucosal injury from gut, liver and plasma [27]. d-Catechin and gallic acid were major component of with effect of hemostasis [28], fried charred Radix et Rhizoma Rhei has more gallic acid and thus hemostasis efficacy was enhanced [29]. pseudoginseng was commonly applied in clinical settings for bleeding, especially bleeding with blood stasis. Dencichine is a special nonprotein amino acid, and the major component of pseudoginseng, which could reduce Prothrombin time (PT), and inhibit platelet aggregation in a dose-dependent manner[30]. Dencichine in a low dose (10 mg·kg^−1^) could stop bleeding via activating endogenous coagulant factor [31]. The local hemostasis effect of Bletilla striata was probably mechanical: jelly of certain thickness was formed by its highly adhesiveness, and thus adhered to the bleeding site. The major effective component of Bletilla striata is mannosan which showed shorter bleeding time than thrombin for minor bleeding (*P*<0.05) [32]. Animal experiments showed that rhizoma bletillae polysaccharide had antiulcer effect, and could enhance gastric mucosal repairmen through inhibition of lipid peroxidation [33]. Radix Ginseng is a common tonifying herb, which supplements spleen Qi and nourishing gastric Yin. According TCM theory, GI patients were Qi and blood deficient, so Qi and Yin should be supplemented at the same time [34]. Cuttlebone derives from a marine animal, comprising calcium carbonate, inhibiting acid and decreasing gastric acid, at the same time, calcium was a kind of coagulation factor, which plays an important part in hemostasis [35].

### 8.3. Supplementing Qi and Hemostasis Formula Has Potential Cardiac Benefit with Minor Interruption of Clopidogrel

The current data showed that 2 MACE occurred in experiment arm including 1 cardiac death; while 6 MACE occurred in control arm including 3 cardiac death. There was not statistical significance between the two arms (*P*>0.05), which means we cannot reckon the MACE incidence was different between two arms. Cardiac event incidence in experiment arm was lowered by 6.95% compared with control, with descending trend and clinical indication. For the 4 patients with cardiac death, the CRUSADE scores were more than 41, and they were aged, kidney dysfunctional, indicating those high risk factors of adverse prognosis after PCI [36, 37].

Our TEG result showed that (1) 8 patients with cardiac events showed low response to clopidogrel, with ADP inhibition of 43.38%±6.53%, which was much lower than those without cardiac events of 49.09%±8.84% (*P*<0.05); MA of ADP was 39.35±5.11mm, higher than those without cardiac events of 34.87±7.26mm(*P*<0.05). Our result was in line with the use of clopidogrel low response in MACE prediction [38]; (2) MA of ADP of control arm at day 30 was increased, inhibition of ADP was decreased, indicating that Pantoprazole Sodium Enteric-Coated Capsule interrupted P450 enzyme; MA of ADP and inhibition pf ADP did not differ after administration of drugs in experiment arm, and the improvement between the two were different significantly, indicating that the effect on P450 from supplementing Qi and hemostasis formula was minor.

According to present guidelines, PPI should be used regularly for those at high risk of GI bleeding [39]. The current consensus is that when combination use of PPI and clopidogrel is needed, the potential interplay as well as patients' general condition should be at physician's discretion [7]. As to choose of PPIs, Pantoprazole and Esomeprazole were preferred to omeprazole [40]. In the current research, supplementing Qi and hemostasis formula showed cardiac benefit, suggesting that this formula only compromised effect of clopidogrel slightly and the underlying mechanism might be associated with the pharmacological effect of the major component.

The effect of supplementing Qi of Astragalus is closely associated with endothelial cell function. Polysaccharides, saponins, Flavone from Astragalus demonstrate cardiovascular effect through various mechanisms [41]. Animal experiments suggested that Astragalus Polysaccharides increased coronary blood flow, reduced infarction size, and the underlying mechanism was associated with Na^+^-K^+^-ATP enzyme inhibition, increase of calcium internal flow, enhancement of myocardial contractile force and antagonism of oxygen free radical [42]. Different from aspirin, Astragalus inhibits platelet through enhancement of prostacyclin (PGI2) and decrease of TXA_2_/ PGI_2_ [43]. Astragaloside of high dose also showed cardiac glycoside action [44]. Rhubarb presents antiischemic effect through antithrombogenesis, endothelial cell protection and lipid modulation. Rhubarb seninside and polysaccharide reduce inner flow of Ca^2+^, block Ca^2+^ channel, and thus decrease Ca^2+^ level, reduce platelet activity, and inhibit platelet aggregation [45]. Rhubarb decreases triglyceride and total cholesterol [46]. Pseudoginseng stops bleeding and activates blood as well. Panax Notoginseng Saponins (PNS) is the major metabolite of pseudoginseng, has shown favorable multiregulation of lipid, hemorheology, and ischemic-reperfusion injury effects [47–49]. Radix Ginseng has improvement on heart failure subsequent to myocardial infarction [50]. Polysaccharide of Radix Radix Ginseng reduced infarction size, and showed protection on heart-lung injury of MI rat [51].

In addition to GI bleeding prevention, supplementing Qi and hemostasis formula showed improvement of palpitation and shortness of breath.

Our data suggested that improvement of TCM syndrome in experiment arm was superior to that of control with statistical significance, which was associated with TCM holistic theory and multitarget, multipathway action of TCM herbs.

In recent years, with evolvement of treatment of some multiple system diseases, modern medicine has realized that many diseases should be considered with the holistic concept, and interplay between various disorders should also be focused on. However, regulation targeted on the preliminary prophylaxis is lacked. We have been looking for a therapy which prevents GI bleeding after PCI with less drug interaction and without cardiac event, but the results were not ideal. The main reason was that regimen targeting gastric mucosa protection and bleeding prevention had its limitation, without the capability to balance bleeding and ischemia.

Holistic concept is a basic character of TCM-the body is wholism, body and nature combined to be another wholism [52]. Differentiation from perspective of holistic concept is the basic advantage of TCM treatment. From the current data, supplementing Qi and hemostasis formula showed hemostasis effect for local gastric mucosa and prophylactic effect of GI bleeding; at the same time, TCM formula presented improvement of the general symptoms, and was superior to control in alleviation of palpitation and breath shortness. This was because the formula focused on the major syndrome of GI bleeding and exerted bidirection regulation of activating blood and hemostasis.

It is well known that TCM herbs have multiple components. This means that a formula comprising 4-6 herbs might consist of hundreds of chemical ingredient, implying that the underlying mechanism possibly involve multiple targets and steps. Supplementing Qi and hemostasis formula enhanced favorable effects, mitigated side-effect as well as prevented GI bleeding effectively. On the contrary, western medicine always focus on gastric mucosa protection, gastric acid inhibition, without overall improvement of ischemic symptoms and long-term prognosis. We reckon that in the multifactor, multigene dominated cardiovascular field, regimen targeted on multistep, multisites is more suitable.

### 8.4. Limitation

Recent reports showed that nonfunctional allelic gene such as CYP2C19 *∗*2, *∗*3 carrier showed lowered level of clopidogrel metabolite, inducing increased remaining platelet activity. As a result, gene polymorphism induced CYP2C19 inactivity was another reason of clopidogrel resistance except drug interaction, and detection of CYP2C19 genotype is correspondingly regarded as a predictor of the sensitivity to clopidogrel [53]. However, a meta-analysis about CYP2C19 genotype, clopidogrel metabolism, and cardiovascular events showed no significant correlation even in western population who carried very low nonfunctional allelic gene [54]. Domestic report also showed the ratio of polymorphism was only 54.5%, while clopidogrel resistance occurred in 61.2% which was much higher [55]. This might be the potential reason of the low specificity of CYP2C19 genotype detection to predicting clopidogrel resistance. In light of the above reasons, the current study did not involve this detection. In addition, the present study was a single-center, randomized controlled trial, with relatively strict inclusion criterion (CRUSADE score≥41 分) and one-year follow-up, so number of participant eligible was extremely limited. Study with enlarged sample size is warranted to further confirm the findings.

## Figures and Tables

**Table 1:** Baseline patient demographics.

	Treatment (N=59)	Control (N=58)	Statistic	*P* value
Age	71.83 ± 8.26	71.14 ± 10.73	0.16(Z)	0.8699
Gender			0.10(X^2^)	0.7518
Male N(%)	22(37.29)	20(34.48)		
Female N(%)	37(62.71)	38(65.52)		
Weight (kg)	65.20 ± 10.95	66.52 ± 11.54	0.33(Z)	0.7451
Height (m)	1.63 ± 0.07	1.62 ± 0.08	-0.66(Z)	0.5083
SBP (mmHg)	130.03 ± 25.04	133.43 ± 23.65	0.54(Z)	0.5911
HR (beat/min)	72.64 ± 13.54	78.45 ± 15.72	2.45(Z)	0.0141

*SBP indicates systolic blood pressure; HR indicates heart rate*.

**Table 2:** Medical history.

	Treatment (N=59)	Control (N=58)	Statistic	*P *value
Hypertension N(%)	45(76.27)	42(72.41)	0.23(X^2^)	0.6328
Type-2 DM N(%)	32(54.24)	33(56.90)	0.08(X^2^)	0.7723
Lipid disorder N(%)	14(23.73)	14(24.14)	0.00(X^2^)	0.9586
Chronic heart dysfunction N(%)	14(23.73)	14(24.14)	0.00(X^2^)	0.9586
Chronic kidney dysfunction N(%)	16(27.12)	15(25.86)	0.02(X^2^)	0.8776
Chronic bronchitis N(%)	5(8.47)	4(6.90)	-(Fisher)	1.0000
Anemia N(%)	3(5.08)	1(1.72)	-(Fisher)	0.6185
Prior cerebrovascular disease N(%)	24(40.68)	15(25.86)	2.89(X^2^)	0.0892
Prior peptic ulcer N(%)	5(8.47)	3(5.17)	-(Fisher)	0.7168
Prior smoking N(%)	20(33.90)	20(34.48)	0.00(X^2^)	0.9469
Prior alcohol intake N(%)	13(22.03)	11(18.97)	0.17(X^2^)	0.6811

**Table 3:** Admitting diagnosis and operation.

	Treatment (N=59)	Control (N=58)	Statistic	*P* value
			0.01(X^2^)	0.9270
Urgen PCI N(%)	30(50.85)	29(50.00)		
Selective PCI N(%)	29(49.15)	29(50.00)		
Admitting diagnosis			0.77(X^2^)	0.8568
STEMI N(%)	13(23.21)	12 (21.43)		
NSTEMI N(%)	14 (25.00)	12 (21.43)		
UAP N(%)	20 (35.71)	20 (35.71)		
SAP N(%)	9 (16.07)	12 (21.43)		
CRUSADE score	56.51 ± 9.73	54.66 ± 9.57	-1.18(Z)	0.2373
PCI puncture access			-(Fisher)	1.0000
Radial artery N(%)	41(69.49)	40(68.97)		
Femoral artery N(%)	18(30.51)	18(31.03)		
Number of diseased vessel			0.37(Z)	0.7099
Single N(%)	19(32.20)	14(24.14)		
Double N(%)	13(22.03)	18(31.03)		
Multiple N(%)	27(45.76)	26(44.83)		
Part of diseased vessel				
LM N(%)	9(15.25)	9(15.52)	0.00(X^2^)	0.9686
AD N(%)	49(83.05)	45(77.59)	0.55(X^2^)	0.4571
CX N(%)	33(55.93)	35(60.34)	0.23(X^2^)	0.6286
RC N(%)	35(59.32)	40(68.97)	1.18(X^2^)	0.2769
Others N(%)	12(20.34)	5(8.62)	3.23(X^2^)	0.0721
Number of stent implantation	2.37 ± 1.38	2.19 ± 1.18	-0.58(Z)	0.5627
IABP implantation	17(28.81)	16(27.59)	0.02(X^2^)	0.8827
Temporary cardiac pacemaker implantation	4(6.78)	3(5.17)	-(Fisher)	1.0000

LM: left main coronary; AD: anterior descending artery; CX: circumflex artery; RC: right coronary artery.

**Table 4:** Comparison between patients with and without ischemic events occurrence.

	Ischemic cardiac event occurred (N=8)	Ischemic cardiac event absent (N=109)	Statistics	*P* value
**Age**	65.50 ± 14.42	71.93 ± 9.01	-1.11(Z)	0.2655
**Gender**			-(Fisher)	0.0245
Male N(%)	6(75.00)	36(33.03)		
Female N(%)	2(25.00)	73(66.97)		
**Weight (kg)**	66.75 ± 16.84	65.79 ± 10.81	-0.40(Z)	0.6889
**Height (m)**	1.66 ± 0.10	1.63 ± 0.07	0.65(Z)	0.5158
**SBP (mmHg)**	140.50 ± 21.39	131.07 ± 24.48	1.29(Z)	0.1967
**HR (beat/min)**	79.38 ± 11.10	75.24 ± 15.13	1.00(Z)	0.3175
**Smoking**	2(25.00)	38(34.86)	-(Fisher)	0.7137
**Alcohol intake**	1(12.50)	23(21.10)	-(Fisher)	1.0000
**Duration of smoking**	20.00 ± 0.00	33.03 ± 15.31	-1.27(Z)	0.2044
**CRUSADE score**	59.88 ± 7.51	55.28 ± 9.75	1.70(Z)	0.0896
**Admitting condition**			-(Fisher)	1.0000
Urgent N(%)	4(50.00)	55(50.46)		
Selective N(%)	4(50.00)	54(49.54)		
**Admitting diagnosis**				0.7641
STEMI N(%)	2(25.00)	24(22.02)		
NSTEMI N(%)	3(37.50)	25(22.94)		
UAP N(%)	2(25.00)	39(35.78)		
SAP N(%)	1(12.50)	21(19.27)		
**Medical history**				
Hypertension N(%)	7(87.50)	80(73.39)	-(Fisher)	0.6779
Type-2 DM N(%)	6(75.00)	59(54.13)	-(Fisher)	0.2971
Lipid disorder N(%)	2(25.00)	26(23.85)	-(Fisher)	1.0000
Chronic heart dysfunction N(%)	2(25.00)	26(23.85)	-(Fisher)	1.0000
Chronic kidney dysfunction N(%)	4(50.00)	27(24.77)	-(Fisher)	0.2056
Chronic bronchitis N(%)	1(12.50)	8(7.34)	-(Fisher)	0.4838
Anemia N(%)	0(0.00)	4(3.67)	-(Fisher)	1.0000
Prior cerebrovascular disease N(%)	1(12.50)	38(34.86)	-(Fisher)	0.2659
Prior peptic ulcer N(%)	1(12.50)	7(6.42)	-(Fisher)	0.4429
**PCI access site**			-(Fisher)	0.7330
Radial artery N(%)	5(62.50)	76(69.72)		
Femoral artery N(%)	3(37.50)	31(28.44)		
Both N(%)	0(0.00)	2(1.83)		
**Number of diseased vessel**			0.39(Z)	0.6975
Single N(%)	3(37.50)	30(27.52)		
Double N(%)	0(0.00)	31(28.44)		
Multiple N(%)	5(62.50)	48(44.04)		
**Part of diseased vessel **				
LM N(%)	3(37.50)	15(13.76)	-(Fisher)	0.1046
AD N(%)	6(75.00)	88(80.73)	-(Fisher)	0.6548
CX N(%)	5(62.50)	63(57.80)	-(Fisher)	1.0000
RC N(%)	7(87.50)	68(62.39)	-(Fisher)	0.2557
Others N(%)	1(12.50)	16(14.68)	-(Fisher)	1.0000
**IABP implantation**	4(50.00)	29(26.61)	-(Fisher)	0.2191
**Temporary cardiac pacemaker implantation **	1(12.50)	6(5.50)	-(Fisher)	0.3992
**Number of stent implantation**	2.50 ± 1.60	2.27 ± 1.26	0.28(Z)	0.7830
**TEG**				
MA_ADP_	39.35 ± 5.11	34.87 ± 7.26	2.09(Z)	0.0366
Inhibition of ADP	43.38 ± 6.53	49.09 ± 8.84	-2.45(Z)	0.0144
Inhibition of AA	44.70 ± 9.63	42.53 ± 9.44	1.17(Z)	0.2413

**Table 5:** TCM syndrome score.

Time		Treatment	Control	Statistics	*P* value^△^
Day 0	Chest pain	4.39 ± 1.23	4.39 ± 1.16	0.07(Z)	0.9444
	Chest tightness	4.67 ± 1.38	4.91 ± 1.37	1.04(Z)	0.2998
	Palpitation	2.35 ± 0.89	2.46 ± 0.78	-0.58(Z)	0.5614
	Shortness of breath	3.16 ± 0.81	3.22 ± 0.77	-0.53(Z)	0.5971
	Debilitation	2.54 ± 0.85	2.74 ± 0.70	-1.44(Z)	0.1503
	Dark and purple lip	0.51 ± 0.32	0.50 ± 0.28	-0.02(Z)	0.9827
	Tongue manifestation	1.31 ± 0.50	1.37 ± 0.43	-0.63(Z)	0.5255
	Pulse manifestation	0.98 ± 0.75	1.03 ± 0.70	-0.32(Z)	0.7489
	TCM syndrome score	19.93 ± 2.63	20.61 ± 2.69	-1.28(Z)	0.1992
Day 7	Chest pain	1.12 ± 1.00*∗*	1.13 ± 1.00*∗*	0.25(Z)	0.8050
	Chest tightness	1.51 ± 0.87*∗*	1.66 ± 0.76*∗*	0.43(Z)	0.6656
	Palpitation	2.21 ± 0.81	2.39 ± 0.75	0.75(Z)	0.4534
	Shortness of breath	3.03 ± 0.87	3.14 ± 0.82	0.75(Z)	0.4534
	Debilitation	2.46 ± 0.88	2.63 ± 0.76	-0.36(Z)	0.7207
	Dark and purple lip	0.45 ± 0.23	0.46 ± 0.21	0.84(Z)	0.4036
	Tongue manifestation	1.22 ± 0.55	1.32 ± 0.47	0.75(Z)	0.4534
	Pulse manifestation	0.88 ± 0.74	0.97 ± 0.72	0.84(Z)	0.4036
	TCM syndrome score	12.88 ± 2.02	13.75 ± 1.99	0.49(Z)	0.6265
Day 30	Chest pain	0.39 ± 0.80*∗*	0.40 ± 0.81*∗*	0.04(Z)	0.9683
	Chest tightness	1.15 ± 1.07*∗*	1.27 ± 1.18*∗*	-0.48(Z)	0.6277
	Palpitation	0.00 ± 0.00*∗*	1.45 ± 0.90*∗*	7.72(Z)	<0.0001
	Shortness of breath	0.47 ± 0.86*∗*	2.16 ± 1.00*∗*	6.48(Z)	<0.0001
	Debilitation	0.32 ± 0.62*∗*	1.99 ± 0.87*∗*	6.22(Z)	<0.0001
	Dark and purple lip	0.04 ± 0.14*∗*	0.31 ± 0.25*∗*	4.54(Z)	<0.0001
	Tongue manifestation	0.13 ± 0.22*∗*	1.13 ± 0.55*∗*	7.55(Z)	<0.0001
	Pulse manifestation	0.09 ± 0.36*∗*	0.95 ± 0.73	5.73(Z)	<0.0001
	TCM syndrome score	2.59 ± 1.87*∗*	9.67 ± 2.02*∗*	7.46(Z)	<0.0001
Day 90	Chest pain	1.12 ± 1.00*∗*	1.16 ± 1.00*∗*	0.23(Z)	0.8206
	Chest tightness	0.78 ± 0.99*∗*	0.73 ± 0.97*∗*	-0.94(Z)	0.3447
	Palpitation	0.48 ± 0.86*∗*	1.23 ± 1.03*∗*	3.59(Z)	0.0003
	Shortness of breath	1.32 ± 0.96*∗*	2.77 ± 0.91*∗*	6.19(Z)	<0.0001
	Debilitation	1.50 ± 0.91*∗*	1.98 ± 1.02*∗*	1.53(Z)	0.1259
	Dark and purple lip	0.23 ± 0.25*∗*	0.26 ± 0.25*∗*	0.79(Z)	0.4272
	Tongue manifestation	0.65 ± 0.65*∗*	1.23 ± 0.51	4.65(Z)	<0.0001
	Pulse manifestation	0.72 ± 0.76*∗*	0.95 ± 0.73	1.37(Z)	0.1695
	TCM syndrome score	6.89 ± 2.17*∗*	10.32 ± 2.16*∗*	4.13(Z)	<0.0001

Note: ^△^ on day 0, P value indicated comparison between the 2 arms; on days 7, 30, and 90, P value indicated the degree of improvement after treatment. *∗* indicated statistically significant difference between pre- and posttreatment.

## Data Availability

The data used to support the findings of this study are available from the corresponding author upon request.
